# Effect of the Wii Sports Resort on the improvement in attention, processing speed and working memory in moderate stroke

**DOI:** 10.1186/s12984-019-0500-5

**Published:** 2019-02-28

**Authors:** Iratxe Unibaso-Markaida, Ioseba Iraurgi, Nuria Ortiz-Marqués, Imanol Amayra, Silvia Martínez-Rodríguez

**Affiliations:** Faculty of Psychology at University of Deusto (Office DARC 1 BIS), 24 Avda. Universidades, 48007 Bilbao, Basque Country Spain

**Keywords:** Moderate stroke, Cognition, Attention, Processing speed, Working memory, Nintendo Wii sports resort

## Abstract

**Background:**

Stroke is the most common neurological disease in the world. After the stroke, some people suffer a cognitive disability. Commercial videogames have been used after stroke for physical rehabilitation; however, their use in cognitive rehabilitation has hardly been studied. The objectives of this study were to analyze attention, processing speed, and working memory in patients with moderate stroke after an intervention with Wii Sports Resort and compared these results with a control group.

**Methods:**

A pre-post design study was conducted with 30 moderate stroke patients aged 65 ± 15. The study lasted eight weeks. 15 participated in the intervention group and 15 belong to the control group. They were assessed in attention and processing speed (TMT-A and B) and working memory (Digit Span of WAIS-III). Parametric and effect size tests were used to analyze the improvement of those outcomes and compared both groups.

**Results:**

At the baseline, there was no difference between TMT-A and B. A difference was found in the scalar score of TMT-B, as well as in Digit Backward Span and Total Digit Task. In TMT-A and B, the intervention group had better scores than the control group. The intervention group in the Digit Forward Span and the Total Digit obtained a moderate effect size and the control group also obtained a moderate effect size in Total Digit. In the Digit scalar scores, the control group achieved better results than the intervention group.

**Conclusions:**

The results on attention, processing speed and working memory improved in both groups. However, according to the effect sizes, the intervention group achieved better results than the control group. In addition, the attention and processing speed improved more than the working memory after the intervention. Although more studies are needed in this area, the results are encouraging for cognitive rehabilitation after stroke.

## Background

Stroke is a really common neurological circulatory disorder, around 795,000 people suffer a new stroke every year and 185,000 are recurrent cases [1]. It is the second most common cause of dementia, death and more than 32% people after stroke suffer from cognitive impairments [2], and the third most common cause of disability which in five years after stroke the disabilities levels increase from 14 to 23% [1]. The after-effects of suffering a stroke can appear on a physical level, such as motor disorders [3, 4], hemiparesis [5], dizziness, vertigo and various sight and speech problems [6]. There can also be cognitive side-effects [7, 8], such as cognitive impairment [9, 10] and various attention disorders [11] on a spatial cognition [12] and behavioral [13] level.

Various studies have been conducted to improve the physical after-effects and to analyze functional capacity through physical activity and motor skills [3, 14, 15], and evidence has been found to suggest that physical activity leads to changes in brain structure [16, 17]. On a physical level, rehabilitation exercises have also been designed to recover the mobility of the affected hands and upper limbs [4, 18], as well as botox (botulinum toxin type A) treatments to improve the spasticity of the affected upper limbs [19].

There has also been research on a psychological level [20] to analyze post-stroke depression [21, 22] and quality of life [2, 23]. To improve the effects on a cognitive level, rehabilitation studies have been conducted to reduce attention deficits [11], aphasia [24] and to work on cognition to improve functional activity [25, 26]. The cognitive after-effects have been studied in the fields of neuropsychology [27] and neurorehabilitation [28, 29]. In neuropsychology, two of the most widely used instruments to measure cognitive abilities such as attention, processing speed and working memory, among others, have been the Trail Making Test [30, 31] and the WAIS Digit Span task [32].

Meanwhile, to decrease the affect-effects of strokes, there have also been studies on the impact of physical activity using commercial videogames, and their use in rehabilitation to control mainly physical consequences [33, 34], such as balance and gait disorders [33, 35] and effects on the upper limbs [36, 37]. However, there is hardly any scientific evidence regarding the use of commercial videogames to do physical activity in order to recover cognition [38, 39]. Hence, the main goal of this study was to evaluate the effect on the cognitive areas of attention, processing speed and working memory in people that have suffered a moderate stroke following an intervention with the Nintendo Wii Sports Resort and compared to a control group who did not receive the intervention with the Nintendo Wii Sport Resort.

## Methods

### Participants

**Fig. 1:**
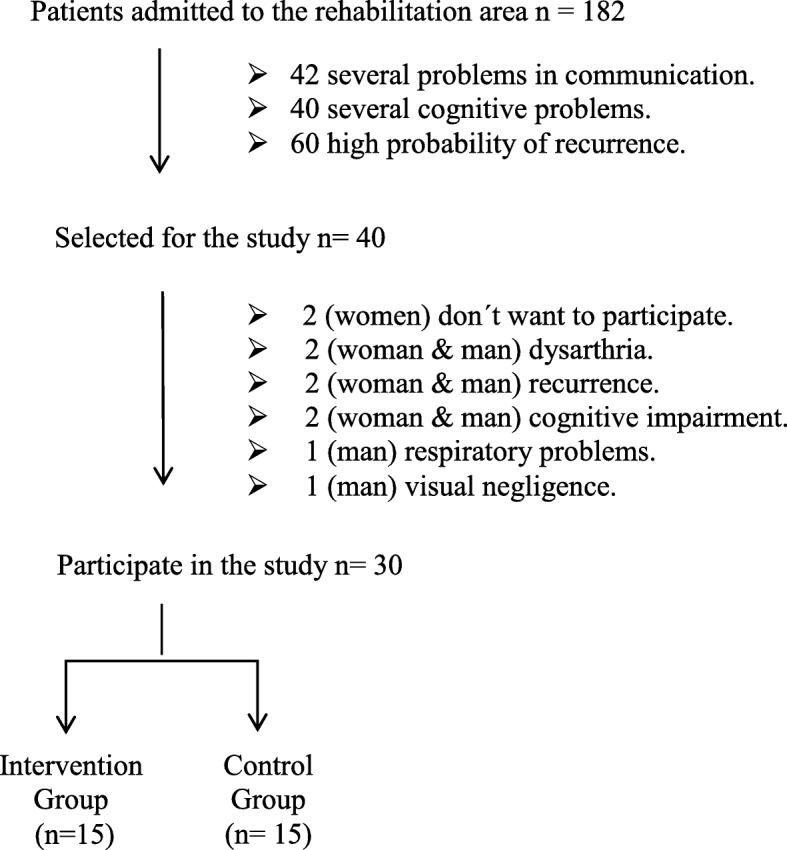
Flow-chart of the sample selection. Only 40 of 182 of the rehabilitation area were selected for the study. 10 participants drop off the study because different causes, two groups were formed by medical professionals. The Intervention Group which realized standard protocol and the intervention with the Nintendo Wii Sports Resort and the Control Group which realized standard protocol and formed the waiting list

The doctors evaluated all patients in the rehabilitation area, 40 of 182 were selected to this study. However, ten patients didn’t take part in the study because different circumstances (didn’t want to participate, recurrences, respiratory problems, etc.). Therefore, the participants were 30 patients in rehabilitation at a specific hospital for such problems that forms part of the public network in Biscay (Basque Country, Spain) (see Fig. 1).

The average age of the participants was 65 (*SD* = 15) and they were mainly men (67%). The inclusion criteria were: being of legal age (+ 18), having been diagnosed as having suffered a moderate stroke using the Oxfordshire Community Stroke Project (OCSP) instrument, a score on the Barthel Scale of between 60 and 90, Mini-Mental (MMSE) with a cut-off of 23 or higher, having suffered a stroke at least 1 month ago, but not more than 1 year ago and those the preservation of the dominant hand in order to conduct the assessment tasks. The exclusion criteria were: patients who don’t follow inclusion criteria and after a stroke who are in unstable clinical condition or are suffering from complications that require active medical treatment, people with comprehension difficulties that prevent them from following verbal instructions, people with dementia or cognitive impairment and people with uncontrolled psychiatric illnesses. Table 1 shows the socio-demographic variables for the entire sample and by intervention groups, revealing that most of them are comparatively similar. There are statistically significant differences in terms of age (*p* = .014; *d* = .99), in that the control group is older than the intervention group (71.55 vs 58.43, respectively); and in educational level (*p* = .040), in that the control group has a higher proportion of participants with a primary education (60%), while the intervention group has a greater proportion, with respect to the expected values, of subjects with higher education (26.7%).

**Table 1:** Sociodemographic characteristics of the sample

		Total Group (*n* = 30)		Intervention Group (*n* = 15)		Control Group (*n* = 15)		Statistics			
		n	%	n	%	n	%	**χ** ^**2**^	df	*p*	p*
*Sex*											
Men		20	66.7	10	66.7	10	66.7	0.00	1	1.00	1.00
Women		10	33.3	5	33.3	5	33.3				
*Current Marital Status*											
Married		15	50.0	9	60.0	6	40.0	2.93	3	.402	.475
Single		10	33.3	5	33.3	5	33.3				
Widowed		2	6.7	0	0.0	2	13.3				
Cohabiting		3	10.0	1	6.7	2	13.3				
*Education*											
Less than primary		1	3.3	1	6.7	0	0.0	10.01	4	.040	.028
Primary		13	43.3	4	26.7	9	60.0				
High school		7	23.3	5	33.3	2	13.3				
A levels		5	16.7	1	6.7	4	26.7				
Graduate studies or Master/PhD		4	13.3	4	26.7	0	0.0				
*Where they live*											
Own home		24	80.0	11	73.3	13	86.7	0.83	1	.361	.651
Relative’s home		6	20.0	4	26.6	2	13.3				
*Who they live with*											
Alone		7	23.3	2	13.3	5	33.3	1.68	1	.195	.390
With others		23	76.7	13	86.7	10	66.7				
*Type of stroke and location*											
Ischemic Right		15	50.0	7	46.7	8	53.3	1.07	3	.785	1.00
Ischemic Left		6	20.0	3	20.0	3	20.0				
Hemorrhagic Right		8	26.7	4	26.7	4	26.7				
Hemorrhagic Left		1	3.3	1	6.7	0	0.0				
		M	SD	M	SD	M	SD	*t*	df	*p*	*d*
*Age*		65.00	15.00	58.43	15.71	71.55	11.37	−2.62	28	.014	.99

Note: **χ**^**2**^: Chi-Square; df: Degree of Freedom; *p*: *p*-value; p*: Exact p-value; M: Mean; SD: Standard Deviation; *t: t-*Student

### Measures

The demographic information was collected using a questionnaire created by the research team.

The Trail Making Test (Part A and Part B) created by Reitan [40], revised in 1992 [41], measures visuomotor speed, attention, motor function, visual scanning, working memory, processing speed and executive functioning. The instrument was created for use with people aged between 16 and 80. The test has two parts. In Part A, the subject has to connect numbers in order from lowest to highest, from 1 to 25; this measures sustained attention and hand-to-eye coordination. Part B adds letters of the alphabet, whereby numbers from 1 to 13 and letters from A to L must be ordered alternately; this assesses the ability to alternate between stimuli and, therefore, mental flexibility. Performance on the test is assessed according to the time taken and errors made. Our study will consider the time taken. There will be no time limit for completing the test, and times will be recorded in seconds.

The Digit Span task is a subtest of the WAIS-III scale created by David Wechsler [42]. It is used to assess attention, resistance to distraction, immediate auditory memory and working memory. It is applicable to people aged between 16 and 89. The test consists of three parts. The first is direct digit-sequencing (DST Forward), in which the participant is given a series of numbers and must repeat them in the same order. The second part involves digits in reverse order, whereby the participant is given a series of numbers and must repeat them backwards (DST Backward). One point is awarded for each correct item and zero points are awarded for each wrong answer. Each of these two tasks is scored between 0 and 16, in such a way that the maximum total score for the Digit Span test is 32 (Total DST). The internal consistency reliability is very high, with values over .90 for all mentioned age ranges, and the test-retest reproducibility is also high (*r* = .75) [42].

### Procedure

A naturalistic pre-post facto study was conducted between June 2016 and February 2017. Informed consent was received from the participants, who were assigned a numerical code to ensure that the data was kept anonymous.

The doctors divided the participants into two groups. There were 15 people in the intervention group, who performed standard protocol and physical activities with the Nintendo Wii Sports Resort game (archery, tennis, golf, bowling, cycling and air sports), 3 days a week for a maximum duration of 30 min per session for 8 weeks. Meanwhile, the other participants formed the control group who performed the standard protocol and were on the waiting list. All of the participants received the service’s same standard protocol in terms of medical and recovery activities which consist on walking around the hospital, sessions of physiotherapist, occupational therapy, logo therapy and neuropsychologist.

The study lasted 8 weeks with 24 sessions. In the first session the psychologist explained the patient the use of the Nintendo Wii and the videogame Sport Resort with the use of the game controller and nunchuck (name of the second game controller). In the others sessions the psychologist was the support for the patients, but they were able to play the games by themselves and in each session patients could play between three or four games (five minutes each game), they took a rest between each game (in total ten minutes’ rest). This study selected these games following a previous study [43].

After the fourth day in the rehabilitation area, a first baseline assessment was conducted to assess cognitive level (attention, processing speed and working memory) using TMT A and B and the WAIS-III Digit Span task. Eight weeks later, the same assessment was carried out for all participants to check their evolution and analyze possible differences between groups and with respect to data for the normative population. After the second assessment, the control group was offered the chance to participate in the same intervention.

### Data analysis

For the description of the socio-demographic data, the Mean (M) and Standard Deviation (SD) were used for the age variable. Frequency and percentages were calculated for the other variables.

With respect to the result variables, both the direct scores for the TMT and the WAIS Digit Span test were transformed into their corresponding scaled scores by age group, on the basis of the normative data offered by the NEURONORMA Project [44, 32]. The direct scores report the time taken for TMT tasks and the number of correct answers in the case of the Digit Span task, in such a way that short completion times and high numbers of correct answers expressed better performance on the respective tests. Meanwhile, the scaled scores offer the position of a subject’s performance with respect to their peers by sex, age and level of education on a scale of 0 to 10, where 10 would be the average value, and where a higher score reflects better performance on the test. For both types of scores, the M and SD are given as characterization statistics, as well as the contrast of differences between means based on a Student’s *t*-test for intergroup (independent *t*-test) and intragroup (*t*-test pairs) comparisons, and the estimation of the size effect by calculating Cohen’s *d* in accordance with the appropriate procedure in each case [45]. For pre-post-facto contrast and contrast between groups for each of the variables of interest, a repeated measures analysis of variance was performed with estimation of the inter- and intra-group and interaction effects. All of the analyses were conducted with the SPSS-V22 statistics program [46].

## Results

### Preliminary analyses

Table 2 shows the performance of the TMT and Digit Span tasks at the baseline for both groups, with no statistically significant differences being observed in the case of TMT tasks, apart from the scaled scores for Part B, where there is a marginal result (*p* = .089; *d* = .64). Differences were observed for the Digit Span task, which are of note when considering the scaled scores for the Backward sub-task (*p* < .001; *d* = 1.40) and Total score (*p* = .004; *d* = 1.15), and marginally so for the Forward sub- task (*p* = .067; *d* = .72).

**Table 2:** *t*-test Before Intervention in TMT, Digit Span Task and their scaled scores (SS)

	Intervention Group (*n* = 15)		Control Group (*n* = 15)		Statistics			
	M	SD	M	SD	*t*	df	*p*	*d*
*TMT*								
Part A	74.60	32.29	82.33	33.38	−0.64	28	.524	.23
Part B	142.07	64.44	152.33	58.46	−0.46	28	.651	.17
Part A (SS)	6.33	2.72	7.67	2.77	−1.33	28	.194	.49
Part B (SS)	8.07	2.43	9.60	2.32	−1.76	28	.089	.64
*Digit Span Task (DST) WAIS- III*								
DST Forward	8.00	2.56	8.13	1.46	−0.17	28	.862	.06
DST Backward	5.73	1.10	6.60	1.92	−1.52	28	.140	.55
Total DST	18.93	4.53	19.67	4.25	−0.46	28	.651	.17
DST Forward (SS)	8.80	3.21	10.87	2.70	−1.91	28	.067	.72
DST Span Backward (SS)	7.73	1.94	11.27	3.15	−3.70	28	.001	1.40
Total DST (SS)	7.53	2.56	10.60	2.77	−3.15	28	.004	1.15

*TMT* Trail Making Test Part A and B, *SS* Scaled Score, *M* Mean, *SD* Standard Deviation, *t t*-Student: *df* degree of freedom, *p p*-value, *d d* Cohen effect size

### Results for TMT

Table 3 shows the data for the TMT, Part A and B, and the scaled scores (A_SS_ and B_SS_, respectively) for both groups before and after the intervention, and the inter- and intra-group effects and the interaction effects. On both parts of the test, the intervention group presents a greater effect size than the control group, for both direct scores (Part A: *d* = 1.17 vs .48, respectively; Part B: *d* = 1.30 vs .68); and the scaled scores (Part A_SS_: *d* = .81 vs .55; Part B_SS_: *d* = 1.65 vs .65). For the different contrasts of the scores for the TMT, statistically significant data is observed for the intragroup effect (*p* ≤ .001), but not for the intergroup or interaction effects.

**Table 3:** TMT, Digit Span, scaled scores (SS) in the intervention and control groups before and after the intervention. ANOVA of repeated measures in comparison of intra- and inter-group scores

	Intervention Group (n = 15)					Control Group (n = 15)										
	Base Line		Eight weeks			Base Line		Eight weeks			Intergroup		Intragroup		Interaction	
	M	SD	M	SD	*d*	M	SD	M	SD	*d*	F	*p*	F	*p*	F	*p*
*TMT*																
Part A	74.60	32.29	54.07	23.22	1.17	82.33	33.38	66.20	23.57	.48	1.17	.288	13.93	.001	0.20	.658
Part B	142.07	64.44	83.27	23.66	1.30	152.33	58.46	112.27	37.34	.68	1.71	.202	26.54	<.001	0.95	.337
Part A (SS)	6.33	2.72	7.93	2.37	.81	7.67	2.77	9.33	2.66	.55	2.63	.116	12.38	.001	0.01	.943
Part B (SS)	8.07	2.43	10.47	2.62	1.65	9.60	2.32	11.27	1.67	.65	2.47	.127	28.25	<.001	0.92	.346
*Digit Span Task (DST) WAIS-III*																
DST Forward	8.00	2.56	9.00	2.90	.49	8.13	1.46	8.40	1.30	.24	0.10	.753	4.50	.043	1.51	.230
DST Backward	5.73	1.10	6.00	1.85	.16	6.60	1.92	7.13	1.99	.25	3.42	.075	1.36	.253	0.15	.700
Total DST	18.93	4.53	20.60	5.41	.56	19.67	4.25	21.93	5.27	.56	0.38	.540	9.28	.005	0.22	.646
DST Forward (SS)	8.80	3.21	10.40	3.42	.50	10.87	2.70	11.40	2.56	.21	2.32	.139	6.32	.018	1.58	.219
DST Backward (SS)	7.73	1.94	8.13	3.18	.16	11.27	3.15	11.80	2.54	.19	17.60	<.001	0.80	.379	0.02	.899
Total DST (SS)	7.53	2.56	8.40	3.14	.43	10.60	2.77	11.67	3.50	.44	9.60	.004	5.66	.024	0.06	.807

*TMT* Trail Making Test Part A and B, *M* Mean, *SD* Standard Deviation, *SS* Scaled Score, *d d* Cohen effect size, *F* ANOVA F-test, *p p*-value

### Results for WAIS- III digit span task

Likewise, Table 3 shows the data for the Digit Span Task (DST). For the intervention group, moderate test-retest effect sizes are presented for DST Backward and Total DST (*d* = .49 and .56, respectively); and for the control group in the case of Total DST (*d* = .56). A statistically marginal intergroup effect is observed for DST Backward (*p* = .075), as well as statistically significant intragroup effects for DST Backward and Total DST (*p* = .043 and .005, respectively), but not for the interaction effects.

With respect to the Digit Span scaled tasks (SS), moderate-high intra-group effects (*p* < .05) are observed for DST Forward (*d* = .50) for the intervention group, and moderate-low effects (*d* = .43 and .44) for Total DST for both groups. Likewise, significant differences are observed for the intergroup effect for DST Backward (*p* < .001) and Total DST (*p* = .004), with better performance being observed in the control group in both cases.

## Discussion

The goal of this study was to describe the changes produced on a cognitive level, to attention and to processing speed as assessed by the TMT (Parts A and B) and to working memory as assessed by the WAIS-III Digit Span task, in subjects who had suffered a moderate stroke, after doing physical activity with the Nintendo Wii (Sports Resort) for a period of 8 weeks, and to compare these results to data from a control group that did no activity with the Wii.

Attention and processing speed improved in this study for both groups. However, the group that performed the intervention with the Nintendo Wii (Sports Resort) improved their performance more than the control group, especially when considering the difference between the effect sizes of the changes found for each group. The analysis of variance presents no evidence that invalidates the null hypothesis of a difference between groups (insignificant effect between groups and for interaction), but does show improved performance for both groups over time. However, sufficiently high effect sizes (*d* >. 80) are observed to consider that a significant change occurred [47, 48]. It is more than likely that the small sample size reduced the statistical power of contrast of the test performed, hence there are limitations for decision-making with regard to the admission of possible differences in the groups’ performance.

Moreover, a paradoxical result is observed with regard to the different TMT scoring formats. The direct scores for both Part A and B show faster completion times among the intervention group than in the control group. Although these are not statistically significant, they would suggest better performance, both at the start and end of the study in this group. However, the standardized scores indicate better performance in the control group than in the intervention group, which contradicts the effect observed for the direct scores. This effect is due to the standardized scores being constructed in accordance with people’s age and level of education, and in our study, differences were observed in terms of both age (higher in the control group) and level of education. This suggests a bias in the allocation of participants to the treatment groups, since said allocation is performed by the doctors responsible for the clinical care, who tended to prefer to use the Wii with younger people on account of believing that they would get the most benefits out of it. However, as commented earlier, the effect sizes of the changes in the two standardized tests were still higher in the intervention group (*d* > .80) than in the control group (between .55 and .65), from which an effect on improved performance associated with use of the Wii can be inferred. In fact, evidence has been found to show that the use of videogames and virtual reality in the same type of population can generate improvements in attention [49]. However, this greater progress in TMT performance may also be due to other variables that were not considered by this study, such as greater age-associated cognitive reserve, which should be observed in future studies.

Meanwhile, for working memory measured by the WAIS-III Digit Span task, and in consideration of the scaled scores, it was observed that in the Digit Span Forward task (DST Forward SS), the intervention group improved more than the control group when considering effect size. However, for DST Backward (DST Backward SS) and Total DST (Total DST SS), it was the control group’s performance that improved. These results contradict the data from the study by Neuronorma Project [32, 44], who proposed weaker performance on this test, depending on the level of education. However, in this study, it is observed that the control group with the lower level of education obtained better results than members of the intervention group who had a university degree or higher. Although the Neuronorma [32] Project found no relationship between this test and age, they do suggest that a decline in performance on this test is to be expected from 50 years of age onwards, an affect that does not agree with this study either, given that the control group was, on average, older than the intervention group. Meanwhile, studies such as that by Kluding [50], involving traditional physical activity and which uses the WAIS-III Digit Span Backward task for evaluation, observe a significant improvement in test performance, while in this study, the intervention using the videogame did not achieve the expected performance.

These results are promising, but inconclusive with regard to the beneficial effects of using the Wii to improve cognitive response among people who have suffered a moderate stroke. There are various limitations that lead us to exercise such caution. The first is related to the weak statistical power caused by working with such a small number of participants. It would be useful for studies to be conducted with a larger number of subjects, but the healthcare conditions themselves (prioritizing effective treatment, the lack of volunteers to take part in evaluation studies, etc.), often lead to the use of limited numbers of participants. One possible solution to increase sample size would be to extend the time for recruitment, but this would mean that the beneficial effects of the intervention could only be demonstrated after a very long time, and the progress of technology would render the resources being used now obsolete. Also conducting multi-center studies could be considered or synthesizing evidence through meta-analysis, but that would imply the need for the right conditions to be established.

A second limitation found of this study is derived from possible bias in the allocation of participants to study groups. This is undoubtedly a major limitation, because it could question the validity of these findings. In this case, considering this effect to have been partially controlled by the use of scaled scores for the TMT and Digit Span tasks, which were constructed precisely on the basis of the variables (age and education level) that were affected by the allocation of participants to groups. The use of completely random allocations and of standardized response variables should be basic goals of any research into healthcare results.

## Conclusion

There is certain evidence of positive results of using the Wii in the recovery of physical functions in people affected by strokes [51]. Those found with respect to the recovery of cognitive functions went according to the results of previous studies [52, 53]. This study draws similar conclusions, and although the evidence is not conclusive, results are observed that seem to indicate a beneficial effect, especially in the areas of attention and processing speed.
